# Interim Effectiveness of Updated 2023–2024 (Monovalent XBB.1.5) COVID-19 Vaccines Against COVID-19–Associated Hospitalization Among Adults Aged ≥18 Years with Immunocompromising Conditions — VISION Network, September 2023–February 2024

**DOI:** 10.15585/mmwr.mm7312a5

**Published:** 2024-03-28

**Authors:** Ruth Link-Gelles, Elizabeth A.K. Rowley, Malini B. DeSilva, Kristin Dascomb, Stephanie A. Irving, Nicola P. Klein, Shaun J. Grannis, Toan C. Ong, Zachary A. Weber, Katherine E. Fleming-Dutra, Charlene E. McEvoy, Omobosola Akinsete, Daniel Bride, Tamara Sheffield, Allison L. Naleway, Ousseny Zerbo, Bruce Fireman, John Hansen, Kristin Goddard, Brian E. Dixon, Colin Rogerson, William F. Fadel, Thomas Duszynski, Suchitra Rao, Michelle A. Barron, Sarah E. Reese, Sarah W. Ball, Margaret M. Dunne, Karthik Natarajan, Erica Okwuazi, Ami B. Shah, Ryan Wiegand, Mark W. Tenforde, Amanda B. Payne

**Affiliations:** ^1^Coronavirus and Other Respiratory Viruses Division, National Center for Immunization and Respiratory Diseases, CDC; ^2^Westat, Rockville, Maryland; ^3^HealthPartners Institute, Minneapolis, Minnesota; ^4^Division of Infectious Diseases and Clinical Epidemiology, Intermountain Health, Salt Lake City, Utah; ^5^Kaiser Permanente Center for Health Research, Portland, Oregon; ^6^Kaiser Permanente Northern California, Oakland, California; ^7^Center for Biomedical Informatics, Regenstrief Institute, Indianapolis, Indiana; ^8^Department of Family Medicine, School of Medicine, Indiana University, Indianapolis, Indiana; ^9^School of Medicine, University of Colorado Anschutz Medical Campus, Aurora, Colorado; ^10^Enterprise Analytics, Intermountain Health, Salt Lake City, Utah; ^11^Immunization Programs, Intermountain Health, Salt Lake City, Utah; ^12^Department of Health Policy and Management, Richard M. Fairbanks School of Public Health, Indiana University, Indianapolis, Indiana; ^13^Department of Pediatrics, School of Medicine, Indiana University, Indianapolis, Indiana; ^14^Department of Biostatistics and Health Data Science, Richard M. Fairbanks School of Public Health, Indiana University, Indianapolis, Indiana; ^15^Department of Epidemiology, Richard M. Fairbanks School of Public Health, Indiana University, Indianapolis, Indiana; ^16^Department of Biomedical Informatics, Columbia University Irving Medical Center, New York, New York; ^17^General Dynamics Information Technology, Falls Church, Virginia; ^18^Influenza Division, National Center for Immunization and Respiratory Diseases, CDC.

**Summary**

**What is already known about this topic?**

In September 2023, CDC’s Advisory Committee on Immunization Practices recommended updated 2023–2024 (monovalent XBB.1.5) COVID-19 vaccination for all persons aged ≥6 months to prevent COVID-19, including severe disease, with optional additional doses for persons with immunocompromising conditions; such persons are at higher risk for severe COVID-19 and might also have reduced immune responses to vaccination.

**What is added by this report?**

Among adults aged ≥18 years with immunocompromising conditions, receipt of an updated COVID-19 vaccine provided increased protection against COVID-19–associated hospitalizations compared with not receiving an updated COVID-19 vaccine. Few persons (18%) in this high-risk study population had received updated COVID-19 vaccine.

**What are the implications for public health practice?**

All persons with immunocompromising conditions should receive updated COVID-19 vaccination and may get additional updated COVID-19 vaccine doses ≥2 months after the last recommended COVID-19 vaccine.

## Abstract

In September 2023, CDC’s Advisory Committee on Immunization Practices recommended updated 2023–2024 (monovalent XBB.1.5) COVID-19 vaccination for all persons aged ≥6 months to prevent COVID-19, including severe disease. As with past COVID-19 vaccines, additional doses may be considered for persons with immunocompromising conditions, who are at higher risk for severe COVID-19 and might have decreased response to vaccination. In this analysis, vaccine effectiveness (VE) of an updated COVID-19 vaccine dose against COVID-19–associated hospitalization was evaluated during September 2023–February 2024 using data from the VISION VE network. Among adults aged ≥18 years with immunocompromising conditions, VE against COVID-19–associated hospitalization was 38% in the 7–59 days after receipt of an updated vaccine dose and 34% in the 60–119 days after receipt of an updated dose. Few persons (18%) in this high-risk study population had received updated COVID-19 vaccine. All persons aged ≥6 months should receive updated 2023–2024 COVID-19 vaccination; persons with immunocompromising conditions may get additional updated COVID-19 vaccine doses ≥2 months after the last recommended COVID-19 vaccine.

## Introduction

On September 12, 2023, CDC’s Advisory Committee on Immunization Practices recommended updated 2023–2024 COVID-19 vaccination with a monovalent XBB.1.5–derived vaccine for all persons aged ≥6 months to prevent COVID-19, including severe disease (*1*). Most persons aged ≥5 years are recommended to receive 1 updated dose. Persons with moderate or severe immunocompromising conditions, who are at higher risk for severe COVID-19 and might have a decreased response to vaccination, have the option to receive additional doses, guided by the clinical judgment of a health care provider and personal preference and circumstances* (*2*). Understanding vaccine effectiveness (VE) among persons with immunocompromising conditions is important to guiding vaccine policy and patient and provider decisions. This analysis estimated effectiveness of updated 2023–2024 COVID-19 vaccines against COVID-19–associated hospitalizations among adults aged ≥18 years with immunocompromising conditions during September 2023–February 2024.

## Methods

Methods for Virtual SARS-CoV-2, Influenza, and Other respiratory viruses Network (VISION) VE analyses have been reported (*3*). VISION is a multisite^†^ electronic health care records (EHR)–based network that utilizes a test-negative design to estimate COVID-19 VE. This analysis included hospitalizations among adults aged ≥18 years with immunocompromising conditions^§^ and who had COVID-19–like illness^¶^ with SARS-CoV-2 molecular testing during the 10 days preceding admission or up to 72 hours after admission. Case-patients were persons who received a positive SARS-CoV-2 test result using a molecular test and received a negative or indeterminate or had an unknown test result for both respiratory syncytial virus and influenza, and control patients were those who received a negative SARS-CoV-2 test result using a molecular test and received a negative influenza test result or had an unknown influenza test result. Nine persons who received >1 updated COVID-19 vaccine dose were included.** Odds ratios (ORs) and 95% CIs were estimated using multivariable logistic regression comparing persons who received an updated COVID-19 vaccine dose with those who did not, irrespective of the number of previous original or bivalent COVID-19 vaccine doses received (if any), among case- and control patients. Regression models were adjusted for age, sex, race and ethnicity, calendar time, and geographic region. VE was calculated as (1 − adjusted OR) × 100%. Analyses were conducted using R software (version 4.3.2; R Foundation). This activity was reviewed by CDC, deemed not research, and was conducted consistent with applicable federal law and CDC policy.^††^ VISION activities were reviewed and approved by the Westat and site institutional review boards. 

## Results

Among 14,586 patients with immunocompromising conditions who were hospitalized with COVID-19–like illness, 1,392 case-patients and 13,194 control patients were included (Table 1). The most common immunocompromising conditions among both case-patients and control patients were solid organ malignancy (36% and 43%, respectively) and other intrinsic immune conditions or immunodeficiency (38% and 35%, respectively). A total of 195 (14%) case-patients had received an updated COVID-19 vaccine dose compared with 2,401 (18%) control patients. VE against COVID-19–associated hospitalization was 38% in the first 7–59 days after receipt of an updated COVID-19 vaccine dose and 34% in the 60–119 days after receipt of an updated dose (Table 2).

**TABLE 1:** Characteristics of hospitalizations among immunocompromised adults aged ≥18 years with COVID-19–like illness, by COVID-19 vaccination status and SARS-CoV-2 test result status — VISION, September 2023–February 2024

Characteristic	Overall, no. (col. %) N = 14,586	SARS-CoV-2 status, no. (column %)		SMD^§^	Vaccination status, no. (row %)			SMD^§^
		Case-patients* (n = 1,392)	Control patients^†^ (n = 13,194)		No updated dose^¶^ (n = 11,990)	Updated dose, 7–59 days earlier (n = 1,381)	Updated dose, 60–119 days earlier (n = 1,215)	
Site**								
HealthPartners	**966 (7)**	91 (7)	875 (7)	0.18	709 (73)	141 (15)	116 (12)	0.49
Intermountain Health	**1,608 (11)**	201 (14)	1,407 (11)		1,358 (84)	125 (8)	125 (8)	
KPNC	**5,790 (40)**	466 (33)	5,324 (40)		4,430 (77)	709 (12)	651 (11)	
KPNW	**863 (6)**	72 (5)	791 (6)		659 (76)	124 (14)	80 (9)	
Regenstrief Institute	**3,541 (24)**	353 (25)	3,188 (24)		3,154 (89)	206 (6)	181 (5)	
University of Colorado	**1,818 (12)**	209 (15)	1,609 (12)		1,680 (92)	76 (4)	62 (3)	
**COVID-19 vaccination status**								
No updated dose^¶^	**11,990 (82)**	1,197 (86)	10,793 (82)	0.12	11,990 (100)	0 (—)	0 (—)	NA
Updated dose, ≥7 days earlier	**2,596 (18)**	195 (14)	2,401 (18)		0 (—)	1,381 (53)	1,215 (47)	
Updated dose, 7–59 days earlier	**1,381 (9)**	100 (7)	1,281 (10)		0 (—)	1,381 (100)	0 (—)	
Updated dose, 60–119 days earlier	**1,215 (8)**	95 (7)	1,120 (8)		0 (—)	0 (—)	1,215 (100)	
**Median age, yrs (IQR)**	**70 (60–79)**	72 (63–80)	70 (60–78)	0.16	69 (59–78)	74 (66–81)	75 (68–82)	0.36
**Age group, yrs**								
18–64	**5,017 (34)**	393 (28)	4,624 (35)	0.15	4,524 (90)	288 (6)	205 (4)	0.43
≥65	**9,569 (66)**	999 (72)	8,570 (65)		7,466 (78)	1,093 (11)	1,010 (11)	
**Female sex**	**7,420 (51)**	669 (48)	6,751 (51)	0.06	6,159 (83)	675 (9)	586 (8)	0.06
**Race and ethnicity**								
Black or African American, non-Hispanic	**1,390 (10)**	110 (8)	1,280 (10)	0.12	1,196 (86)	113 (8)	81 (6)	0.14
White, non-Hispanic	**10,008 (69)**	1,022 (73)	8,986 (68)		8,126 (81)	996 (10)	886 (9)	
Hispanic or Latino	**1,620 (11)**	127 (9)	1,493 (11)		1,378 (85)	135 (8)	107 (7)	
Other, non-Hispanic^††^	**1,419 (10)**	121 (9)	1,298 (10)		1,157 (82)	130 (9)	132 (9)	
Unknown^§§^	**149 (1)**	12 (1)	137 (1)		133 (89)	7 (5)	9 (6)	
**No. of chronic medical condition categories^¶¶^ in addition to immunocompromising conditions**								
0	**555 (4)**	31 (2)	524 (4)	0.13	495 (89)	33 (6)	27 (5)	0.17
1	**1,310 (9)**	144 (10)	1,166 (9)		1,138 (87)	91 (7)	81 (6)	
2	**2,681 (18)**	267 (19)	2,414 (18)		2,204 (82)	255 (10)	222 (8)	
3	**4,115 (28)**	400 (29)	3,715 (28)		3,307 (80)	404 (10)	404 (10)	
4	**3,378 (23)**	333 (24)	3,045 (23)		2,741 (81)	354 (10)	283 (8)	
≥5	**2,547 (17)**	217 (16)	2,330 (18)		2,105 (83)	244 (10)	198 (8)	
Chronic respiratory condition***	**6,192 (42)**	550 (40)	5,642 (43)	0.07	5,012 (81)	628 (10)	552 (9)	0.07
**Type of immunocompromising condition^†††^**								
Solid organ malignancy	**6,185 (42)**	500 (36)	5,685 (43)	0.15	5,052 (82)	600 (10)	533 (9)	0.03
Hematologic malignancy	**2,124 (15)**	241 (17)	1,883 (14)	0.08	1,699 (80)	234 (11)	191 (9)	0.06
Rheumatologic or inflammatory disorder	**3,684 (25)**	411 (30)	3,273 (25)	0.11	3,025 (82)	345 (9)	314 (9)	0.01
Other intrinsic immune condition or immunodeficiency	**5,140 (35)**	525 (38)	4,615 (35)	0.06	4,304 (84)	445 (9)	391 (8)	0.08
Organ or stem cell transplant	**1,191 (8)**	162 (12)	1,029 (8)	0.13	974 (82)	122 (10)	95 (8)	0.02
HIV/AIDS	**315 (2)**	17 (1)	298 (2)	0.08	258 (82)	34 (11)	23 (7)	0.02
**ICU admission**	**3,386 (23)**	283 (20)	3,103 (24)	0.08	2,871 (85)	298 (9)	217 (6)	0.10
**Receipt of invasive mechanical ventilation**								
Yes	**1,467 (10)**	117 (8)	1,350 (10)	0.07	1,271 (87)	107 (7)	89 (6)	0.27
No	**10,967 (75)**	1,059 (76)	9,908 (75)		8,788 (80)	1,155 (11)	1,024 (9)	
Unknown	**2,152 (15)**	216 (16)	1,936 (15)		1,931 (90)	119 (6)	102 (5)	
**In-hospital death^§§§^**	**1,479 (10)**	112 (8)	1,367 (10)	0.08	1,249 (84)	125 (8)	105 (7)	0.05
**Month and year of COVID-19–like illness hospitalization**								
Sep 2023	**931 (6)**	68 (5)	863 (7)	0.20	931 (100)	0 (—)	0 (—)	1.10
Oct 2023	**3,045 (21)**	247 (18)	2,798 (21)		2,901 (95)	144 (5)	0 (—)	
Nov 2023	**3,015 (21)**	285 (20)	2,730 (21)		2,503 (83)	496 (16)	16 (1)	
Dec 2023	**3,394 (23)**	394 (28)	3,000 (23)		2,596 (76)	482 (14)	316 (9)	
Jan 2024	**3,214 (22)**	337 (24)	2,877 (22)		2,334 (73)	223 (7)	657 (20)	
Feb 2024	**987 (7)**	61 (4)	926 (7)		725 (73)	36 (4)	226 (23)	
**SARS-CoV-2 JN.1 lineage predominant period** ^¶¶¶^	**5,089 (35)**	512 (37)	4,577 (35)	0.04	3,732 (73)	346 (7)	1,011 (20)	0.69

**Abbreviations:** ICU = intensive care unit; KPNC = Kaiser Permanente Northern California; KPNW = Kaiser Permanente Northwest; NA = not applicable; SMD = standardized mean or proportion difference; VISION = Virtual SARS-CoV-2, Influenza, and Other respiratory viruses Network.

* Patient received a positive SARS-CoV-2 test result using a molecular test and received a negative or indeterminate test result or had an unknown test result for both respiratory syncytial virus and influenza.

^†^ Patient received a negative SARS-CoV-2 test result using a molecular test and received a negative influenza test result or had an unknown influenza test result.

^§^ A larger SMD indicates a larger difference in variable distributions between hospitalizations for vaccinated versus unvaccinated patients, or for patients who received a positive SARS-CoV-2 test result versus patients who received a negative SARS-CoV-2 test result. For mRNA COVID-19 vaccination status, a single SMD was calculated by averaging the absolute SMDs obtained from pairwise comparisons of each vaccinated category versus unvaccinated. Specifically, SMD was calculated as the average of the absolute value of the SMDs for 1) updated dose, 7–59 days earlier versus no updated dose; and 2) updated dose, 60–119 days earlier versus no updated dose.

^¶^ The “no updated dose” group included all eligible persons who did not receive an updated COVID-19 vaccine dose, regardless of number of previous (i.e., original monovalent and bivalent) doses (if any) received.

** Date ranges of hospitalizations by site: HealthPartners (September 21, 2023–February 17, 2024), Intermountain Health (September 21, 2023–February 17, 2024), KPNC (September 21, 2023–February 17, 2024), KPNW (September 21, 2023–February 17, 2024), Regenstrief Institute (September 21, 2023–February 13, 2024), and University of Colorado (September 21, 2023–February 4, 2024).

^††^ “Other, non-Hispanic” race persons reporting non-Hispanic ethnicity and any of the following options for race: American Indian or Alaska Native, Asian, Native Hawaiian or other Pacific Islander, other races not listed, and multiple races; because of small numbers, these categories were combined.

^§§^ “Unknown” includes persons with missing race and ethnicity in their electronic health records.

^¶¶^ Underlying condition categories included pulmonary, cardiovascular, cerebrovascular, musculoskeletal, neurologic, hematologic, endocrine, renal, and gastrointestinal. All persons in the analysis had one or more immunocompromising condition.

*** Chronic respiratory condition was defined using *International Classification of Diseases, Tenth Revision* discharge codes for asthma, chronic obstructive pulmonary disease, cystic fibrosis, or other lung disease.

^†††^ Persons included in the analysis might have one or more immunocompromising conditions; therefore, column totals might add to more than 100%.

^§§§^ In-hospital death was defined as death while hospitalized within 28 days after admission.

^¶¶¶^ The JN.1 predominant period was considered to have started December 24, 2023.

**TABLE 2:** Effectiveness of updated 2023–2024 (monovalent XBB.1.5) COVID-19 vaccination against laboratory-confirmed COVID-19–associated hospitalization among immunocompromised adults aged ≥18 years — VISION, September 2023–February 2024

COVID-19 vaccination dosage pattern	Total	Positive SARS-CoV-2 test result, no. (%)	Median interval since last dose, days (IQR)	Unadjusted VE, %* (95% CI)	Adjusted VE, %^†^ (95% CI)
No updated dose^§^ (Ref)	**11,990**	1,197 (10)	587 (381–766)	**Ref**	**Ref**
Received updated dose	**2,596**	195 (8)	56 (32–81)	**27 (14–37)**	**36 (25–46)**
7–59 days earlier	**1,381**	100 (7)	34 (21–46)	**30 (13–43)**	**38 (23–50)**
60–119 days earlier	**1,215**	95 (8)	83 (71–98)	**24 (5–38)**	**34 (16–47)**

**Abbreviations:** Ref = referent group; VE = vaccine effectiveness; VISION = Virtual SARS-CoV-2, Influenza, and Other respiratory viruses Network.

* VE was calculated as (1 – odds ratio) × 100%, with odds ratios calculated using logistic regression.

^†^ The odds ratio was adjusted for age, sex, race and ethnicity, geographic region, and calendar time (days since January 1, 2021).

^§^ The “no updated dose” group included all eligible persons who did not receive an updated COVID-19 vaccine dose, regardless of number of previous (i.e., original monovalent and bivalent) doses (if any) received.

## Discussion

In this multisite analysis among adults with immunocompromising conditions during September 2023–February 2024, receiving an updated 2023–2024 COVID-19 vaccine dose provided additional protection against COVID-19–associated hospitalizations, compared with not receiving an updated vaccine dose. Effectiveness estimates in this report were slightly lower than those in a recently published analysis from VISION and another CDC VE network showing COVID-19 VE against COVID-19-associated hospitalizations in adults without immunocompromising conditions was approximately 50%, but this report includes the analysis of an additional month of data compared with the previous report (*3*). However, lower COVID-19 VE among adults with immunocompromising conditions compared with adults without immunocompromising conditions has been previously reported (*4*,*5*); persons with moderate or severe immunocompromising conditions are at higher risk for severe COVID-19 and might have decreased response to vaccination (*2*).

Relatively few persons in this analysis had received an updated COVID-19 vaccine dose, despite those with immunocompromising conditions being at higher risk for severe COVID-19. For example, among those with an organ or stem cell transplant, a group known to be at particularly high risk for severe COVID-19 (*6*), only 18% had received an updated dose, representing a missed opportunity to prevent severe COVID-19.

### Limitations

The findings in this report are subject to at least two limitations. First, the use of selected discharge diagnoses as surrogates for presumed immunocompromise status and the absence of medication and other relevant data might have led to misclassification of immunocompromise status, which might have biased estimated VE in either direction. Second, immunocompromising conditions are heterogeneous and likely to create differential risk for severe COVID-19, as well as differential response to vaccination (*2*). This analysis did not have statistical power to estimate VE by individual risk group or for those receiving more than one dose of the updated COVID-19 vaccine; however, CDC will continue to monitor VE in these groups.  In addition, this analysis is subject to limitations similar to those in previous VISION VE analyses, including the potential that case-patients might have been hospitalized for reasons other than COVID-19, potential misclassification of vaccination status, no accounting for previous infection status, and potential residual confounding (*3*).

### Implications for Public Health Practice

Receipt of an updated COVID-19 vaccine dose provided increased protection against COVID-19–associated hospitalization among adults with immunocompromising conditions compared with no receipt of an updated dose. CDC will continue to monitor VE of updated COVID-19 vaccines in populations at high risk, including those with immunocompromising conditions. All persons aged ≥6 months should receive updated 2023–2024 COVID-19 vaccination; persons with immunocompromising conditions may get additional updated COVID-19 vaccine doses ≥2 months after the last recommended COVID-19 vaccine.

## Footnotes

*
https://www.cdc.gov/vaccines/covid-19/clinical-considerations/interim-considerations-us.html

† Sites from the CDC-funded VISION network that contributed data for this analysis were HealthPartners (Minnesota and Wisconsin), Intermountain Health (Utah), Kaiser Permanente Northern California (California), Kaiser Permanente Northwest (Oregon and Washington), Regenstrief Institute (Indiana), and University of Colorado (Colorado).

§ Immunocompromising conditions were obtained from *International Classification of Diseases, Tenth Revision* (ICD-10) discharge codes. The specific codes used were hematological malignancy: C81.*, C82.*, C83.*, C84.*, C85.*, C86.*, C88.*, C90.*, C91.*, C92.*, C93.*, C94.*, C95.*, C96.*, D46.*, D61.0*, D61.2, D61.9, D70.0, and D71.*; solid malignancy: C00.*, C01.*, C02.*, C03.*, C04.*, C05.*, C06.*, C07.*, C08.*, C09.*, C10.*, C11.*, C12.*, C13.*, C14.*, C15.*, C16.*, C17.*, C18.*, C19.*, C20.*, C21.*, C22.*, C23.*, C24.*, C25.*, C26.*, C27.*, C28.*, C29.*, C30.*, C31.*, C32.*, C33.*, C34.*, C35.*, C36.*, C37.*, C38.*, C39.*, C40.*, C41.*, C42.*, C43.*, C44.*, C45.*, C46.*, C47.*, C48.*, C49.*, C50.*, C51.*, C52.*, C53.*, C54.*, C55.*, C56.*, C57.*, C58.*, C59.*, C60.*, C61.*, C62.*, C63.*, C64.*, C65.*, C66.*, C67.*, C68.*, C69.*, C70.*, C71.*, C72.*, C73.*, C74.*, C75.*, C76.*, C77.*, C78.*, C79.*, C7A.*, C7B.*, C80.*, Z51.0, Z51.1*, and C4A.*; transplant: T86.0*, T86.1*, T86.2*, T86.3*, T86.4*, T86.5*, T86.81*, T86.85*, D47.Z1, Z48.2.*, Z94.*, and Z98.85; rheumatologic/inflammatory disorders: D86.*, E85.1, E85.2, E85.3, E85.4, E85.8*, E85.9, G35.*, J67.9.*, L40.54, L40.59, L93.0.*, L93.2.*, L94.*, M05.*, M06.*, M07.*, M08.*, M30.*, M31.3*, M31.5*, M32.*, M33.*, M34.*, M35.3*, M35.8*, M35.9*, M46.*, and T78.40*; other intrinsic immune condition or immunodeficiency: D27.9, D72.89, D80.*, D81.0, D81.1, D81.2, D81.4, D81.5, D81.6, D81.7, D81.8*, D81.9, D82.*, D83.*, D84.*, D87.89, D89.0, D89.1, D89.3, D89.4*, D89.8*, D89.9, K70.3*, K70.4*, K72.*, K74.3, K74.4, K74.5, K74.6, N04.*, R18.0; HIV: B20.*, B21.*, B22.*, B23.*, B24.*, B97.35, O98.7*, and Z21*. All ICD-10 codes with * include all child codes under the specific parent code.

¶ COVID-19–like illness diagnoses were obtained from ICD-10 discharge codes. The specific codes used were COVID-19 pneumonia: J12.81 and J12.82; influenza pneumonia: J09.X1, J10.0, J10.00, J10.01, J10.08, J11.0, J11.00, and J11.08; other viral pneumonia: J12*; bacterial and other pneumonia: J13, J14, J15*, J16*, J17, and J18*; influenza disease: J09*, J10.1, J10.2, J10.8*, J11.1, J11.2, and J11.8*; acute respiratory distress syndrome: J80; chronic obstructive pulmonary disease with acute exacerbation: J44.1; asthma acute exacerbation: J45.21, J45.22, J45.31, J45.32, J45.41, J45.42, J45.51, J45.52, J45.901, and J45.902; respiratory failure: J96.0*, J96.2*, and R09.2; other acute lower respiratory tract infections: B97.4, J20*, J21*, J22, J40, J44.0, J41*, J42, J43*, J47*, J85, J85.0, J85.1, J85.2, J85.3, and J86*; acute and chronic sinusitis: J01* and J32*; acute upper respiratory tract infections: J00*, J02*, J03*, J04*, J05*, and J06*; acute respiratory illness signs and symptoms: R04.2, R05, R05.1, R05.2, R05.4, R05.8, R05.9, R06.00, R06.02, R06.03, R06.1, R06.2, R06.8, R06.81, R06.82, R06.89, R07.1, R09.0*, R09.1, R09.2, R09.3, and R09.8*; acute febrile illness signs and symptoms: R50*, R50.81, and R68.83; acute nonrespiratory illness signs and symptoms: M79.10, M79.18, R19.7, R43*, R51.9, R65*, R53.81, R53.83, R57.9, R41.82, R40.0, R40.1, R53.1, R11.0, R11.10, R11.11, R11.15, R11.2, R21*, R10.0, R10.1*, R10.2, R10.3*, R10.81*, R10.84, and R10.9; respiratory failure, unspecified: J96.9*; febrile convulsions: R56.0; viral and respiratory diseases complicating pregnancy, childbirth, and puerperium: O98.5*, O98.8*, O98.9*, and O99.5*. All ICD-10 codes with * include all child codes under the specific parent code. One VISION site, representing 33% of case-patients, did not include the following codes in its definition: B97.4, J96.9*, O98.5*, O98.8*, O98.9*, O99.5*, and R56.0.

** The Advisory Committee on Immunization Practices recommendations allow optional additional doses for persons with moderate or severe immunocompromise. Because only nine persons in participating sites received >1 updated COVID-19 vaccine dose, statistical power to estimate VE separately in this group was insufficient.

†† 45 C.F.R. part 46.102(l)(2), 21 C.F.R. part 56; 42 U.S.C. Sect. 241(d); 5 U.S.C. Sect. 552a; 44 U.S.C. Sect. 3501 et seq.

## References

[1] Regan JJ, Moulia DL, Link-Gelles R, Use of updated COVID-19 vaccines 2023–2024 formula for persons aged ≥6 months: recommendations of the Advisory Committee on Immunization Practices—United States, September 2023. MMWR Morb Mortal Wkly Rep 2023;72:1140–6. 10.15585/mmwr.mm7242e1 37856366 PMC10602621

[2] Lee ARYB, Wong SY, Chai LYA, Efficacy of covid-19 vaccines in immunocompromised patients: systematic review and meta-analysis. BMJ 2022;376:e068632. 10.1136/bmj-2021-068632 35236664 PMC8889026

[3] DeCuir J, Payne AB, Self WH, ; CDC COVID-19 Vaccine Effectiveness Collaborators. Interim effectiveness of updated 2023–2024 (monovalent XBB.1.5) COVID-19 vaccines against COVID-19–associated emergency department and urgent care encounters and hospitalization among immunocompetent adults aged ≥18 years—VISION and IVY networks, September 2023–January 2024. MMWR Morb Mortal Wkly Rep 2024;73:180–8. 10.15585/mmwr.mm7308a5 38421945 PMC10907041

[4] Britton A, Embi PJ, Levy ME, Effectiveness of COVID-19 mRNA vaccines against COVID-19–associated hospitalizations among immunocompromised adults during SARS-CoV-2 Omicron predominance—VISION network, 10 states, December 2021–August 2022. MMWR Morb Mortal Wkly Rep 2022;71:1335–42. 10.15585/mmwr.mm7142a4 36264840 PMC9590295

[5] Link-Gelles R, Weber ZA, Reese SE, Estimates of bivalent mRNA vaccine durability in preventing COVID-19–associated hospitalization and critical illness among adults with and without immunocompromising conditions—VISION network, September 2022–April 2023. MMWR Morb Mortal Wkly Rep 2023;72:579–88. 10.15585/mmwr.mm7221a3 37227984 PMC10231940

[6] Fisher AM, Schlauch D, Mulloy M, Outcomes of COVID-19 in hospitalized solid organ transplant recipients compared to a matched cohort of non-transplant patients at a national healthcare system in the United States. Clin Transplant 2021;35:e14216. 10.1111/ctr.14216 33406279 PMC7883091

